# Homocysteine as a Risk Factor for Hypertension: A 2-Year Follow-Up Study

**DOI:** 10.1371/journal.pone.0108223

**Published:** 2014-10-13

**Authors:** Yixuan Wang, Shuohua Chen, Tao Yao, DongQing Li, YanXiu Wang, YuQing Li, ShouLing Wu, Jun Cai

**Affiliations:** 1 Department of Cardiology, Beijing Chao-Yang Hospital, Capital Medical University, Chaoyang District, Beijing, China; 2 Health Department of Kailuan Group, Tangshan, China; 3 Linxi Hospital, Affiliated Kailuan General Hospital, Hebei United University, Tangshan, China; 4 Department of Cardiology, The Kailuan General Hospital, Hebei United University, Tangshan, China; 5 Department of Rheumatology and Immunology, The Kailuan General Hospital, Hebei United University, Tangshan, China; University of Pécs Medical School, Hungary

## Abstract

Homocysteine (Hcy) is regarded as a risk factor for hypertension, but research on the causal relationship between Hcy and hypertension is limited. In the present study, we prospectively tracked the blood pressure progression of a normotensive population with different Hcy levels over a 2-year period. The incidence of hypertension with increasing Hcy quartiles produced an approximately U-shaped curve, with significance in males. Compared with the third quartile, the risk of hypertension in the first and second quartiles was increased by 1.55 (95% confidence interval [CI] 1.154–2.081) fold and 1.501 (95% CI 1.119–2.013) fold, respectively, with the increase being more significant in males. In conclusion, Hcy is related to hypertension incidence with the results approximating an U-shaped curve. Low Hcy levels might also increase the risk of hypertension.

## Introduction

Hypertension is regarded as a modifiable risk factor for cardiovascular disease, and is increasing as an economic burden worldwide. Multiple intervention mechanisms are important for controlling and preventing the disease [1], but its etiology has not been fully elucidated. Recently, hyper-homocysteinemia (HHcy), generally defined as plasma homocysteine (Hcy)≥10 µmol/L, has been regarded as a new risk factor related to hypertension [2]–[5].

Hcy is an intermediate sulfur-containing amino acid in the metabolism of methionine. It is recycled either by trans-sulfuration to cysteine or by remethylation to methionine, and is mainly cleared through the kidneys [6], [7]. Numerous nutritional deficiencies (folate, and vitamins B12 and B6 as cofactors of methionine metabolism), genetic variation (methylene tetrahydrofolate reductase), drugs (phenytoin, carbamazepine), or diseases (renal insufficiency) affect Hcy metabolism and influence serum Hcy levels [8]. HHcy causes vascular dysfunction mainly through its oxidative effects, which could reduce vasodilators like nitric oxide as well as promote extracellular matrix accumulation and smooth muscle cell proliferation, which could lead to vascular constriction and stiffness [9], [10].

Epidemiological studies demonstrated similar distributions of HHcy and hypertension, and both were related to an increased risk of cardiovascular events [3], [11]. In a large epidemiological study (NHANES III) [12], each 5 µmol/L increase in plasma Hcy levels was associated with an increase in systolic (SBP) and diastolic blood pressure (DBP) of 0.7 and 0.5 mmHg, respectively, in men, and 1.2 and 0.7 mmHg, respectively, in women. However, the effect of Hcy-lowering interventions seemed to be paradoxical in the hypertensive population. Nutritional supplements could lower Hcy levels in most studies, but this was not always related to blood pressure [13], [14]. These results identified the need for prospective studies to illustrate whether there is direct association between Hcy and hypertension, or if these two factors just loosely coexist.

To investigate the causal relationship between Hcy and hypertension based on the Kailuan Study (register number: ChiCTR-TNC-11001489), we prospectively tracked the blood pressure progression of a non-hypertensive population with different Hcy levels for 2 years. The incidence of hypertension and blood pressure progression was investigated, and the risk of incident hypertension by Hcy was evaluated.

## Materials and Methods

The study was performed according to the guidelines outlined in the Declaration of Helsinki and was jointly approved by the Ethics Committee of Kailuan General Hospital, Beijing Chaoyang Hospital, and TianTan Hospital. Written informed consent was obtained from all participants.

### Study population

According to the sex and age distribution of the country populace aged 40 years and older in the 2005 1% sampling demographic census, subjects in this study were randomly drawn from the staff in the Kailuan group who participated in the 2010–2011 physical examinations biannually. In the observational cohort of 5440 cases, there were 2836 cases that met the inclusion criteria (SBP<140 mmHg and DBP<90 mmHg) of the study. For a variety of reasons, 315 cases failed to participate in the 2012–2013 physical examinations. No Hcy was detected in 36 cases, and 13 cases had a history of hypertension, but their blood pressure values were missing and these cases were excluded. Finally, valid data from 2472 cases were included in the statistical analysis. The elimination criteria included SBP≥140 mmHg, DBP≥90 mmHg, or taking antihypertensive medication at the time of the 2010–2011 physical examination, missing the 2012–2013 physical examination, Hcy data missing, cognitive or physical disability, brain apoplexy (exception of lacunar infarction), transient ischemic attack, myocardial infarction, past history of hypertension, and death during follow-up. All subjects provided informed consent for this study.

### Data Collection and Measurements

Epidemiological characteristics were recorded as previously described [15]. Right brachial blood pressure was measured using a mercury sphygmomanometer while the subject was in a supine position after resting for 15 minutes, and the subject was not permitted to have coffee or tea within 30 minutes prior to the measurement. Phases I and V of the Korotkoff sounds were considered as SBP and DBP, respectively. Three measurements taken at 1–2 minute intervals were averaged. High blood pressure was diagnosed according to JNC VI criteria (SBP≥140 mmHg, DBP≥90 mmHg, or use of antihypertensive medication), and non-hypertensive subjects were classified into the optimal blood pressure group (SBP<120 mmHg and DBP<80 mmHg), normal blood pressure group (SBP: 120–129 mmHg and DBP 80–84 mmHg), and high-normal blood pressure group (SBP: 130–139 mmHg and DBP 85–89 mmHg). Smoking was defined as smoking more than one cigarette a day for at least one year, and also as being an ex-smoker. Alcohol consumption was defined as intake of any alcoholic drinks, and excluded ex-drinkers. Exercise was defined as aerobic exercise for ≥30 min ≥3 times per week, including walking, jogging, team sports, and so on. Dietary salt intake was classified as salty (>12 g/d), normal (6∼12 g/d), and light (<6 g/d). The study end point was occurrence of hypertension or blood pressure that had progressed more than one stage at the 2012–2013 physical examination.

Venous blood samples (5 mL) were obtained from the antecubital vein in the morning after a 12-h fast. Blood was collected into vacutainers (BD) and placed at room temperature for 20–30 min before centrifugation at 3,000 rpm for 15 min. Serum total cholesterol (TC), triglyceride (TG), fasting blood glucose (FBG), high-density lipoprotein (HDL), low-density lipoprotein (LDL), uric acid (UA), and creatinine (CR) levels were measured. Hcy was measured by fluorescence polarization immunoassay (FPIA, Abbott) on an automated immunoassay analyzer (Abbott). All measurements were carried out in a certified laboratory, and quality control was conducted with each batch. All participants were followed for two years, and all biochemical parameters and blood pressure measurements were collected at the beginning of the study and at each follow-up visit.

### Statistical analysis

Database construction was as previously described [15]. Continuous variables were expressed as mean ± standard deviation (SD), or median (interquartile range [IQR]). Comparisons between groups were done by *t*-test or analysis of variance (ANOVA). The distribution of Hcy levels was determined in the whole population and in each sex after dividing into quartiles according to the lg(−Hcy). Logistic regression was performed to study the risk of hypertension and blood pressure progression by Hcy. Data were analyzed using SPSS software version 13.0. Values of P<0.05 were considered statistically significant.

## Results

### Baseline Clinical Characteristics of the Study population

The cohort of 2427 subjects consisted of 1215 males (49.2%), had an average age of 51.26 years, and average Hcy levels of 12.3 µmol/L at the 2010–2011 physical examination. There were 840 subjects (34.0%) in the optimal blood pressure group, 943 (38.1%) in the normal group, and 689 (27.9%) in the high-normal blood pressure groups. The baseline clinical characteristics are shown in Table 1.

**Table 1:** Baseline demographic and clinical characteristics of the study population at the 2010–2011 physical examinations.

Clinical characteristics	Values
**Males n (%)**	1215 (49.2)
**Mean age ± SD, years**	51.26±9.63
**Mean BMI ± SD, Kg/m2**	24.23±2.94
**Mean waistline, ± SD; mm**	83.24±9.17
**Mean FBG ± SD, mmol/L**	5.35±1.19
**Mean TC ± SD, mmol/L**	4.98±0.92
**Mean LDL-C ± SD, mmol/L**	1.67±0.47
**Mean HDL-C ± SD, mmol/L**	2.6±0.71
**Median TG (95% CI), mmol/L**	1.2 (0.86,1.74)
**Mean CR ± SD, umol/L**	71.27±17.39
**Mean UA± SD, µmol/L**	271.48±81.83
**Mean hs-CRP, mg/L**	0.85 (0.47,1.70)
**Mean DBP ± SD, mmHg**	117.9±11.43
**Mean SBP ± SD, mmHg**	76.75±7.01
**Optimal group, n (%)**	840 (34.0)
**Normal group, n (%)**	943 (38.1)
**High-normal group, n (%)**	689 (27.9)
**Mean HR ± SD, bpm**	68.91±9.68
**Smoking n (%)**	803 (32.5)
**Drinking n (%)**	647 (26.2)
**Exercise n (%)**	789 (31.9)
**Salty n (%)**	442 (17.9)
**Median Hcy, µmol/L (95% CI)**	12.3 (8.6,17.5)
**Hcy >16 µmol/L, n (%)**	746 (30.2)

FBG: Fast blood glucose; TG: Triglyceride; TC: Total cholesterol; HDL-C: High-density lipoprotein cholesterol; LDL-C: Low-density lipoprotein cholesterol; BMI: Body mass index; UA: uric acid; CR: creatinine; hs-CRP: high sensitivity C-reactive protein; Hcy: homocysteine.

### Incidence of Hypertension and Blood Pressure Progression by Different Hcy Quartiles

**Figure 1:**
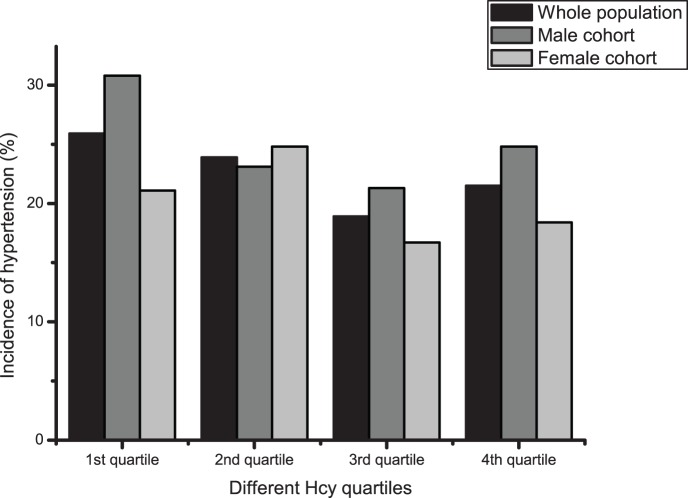
Incidence of hypertension within different Hcy quartiles. Black bar: Whole population; Dark gray bar: Male cohort; Light grey bar: Female cohort.

**Figure 2:**
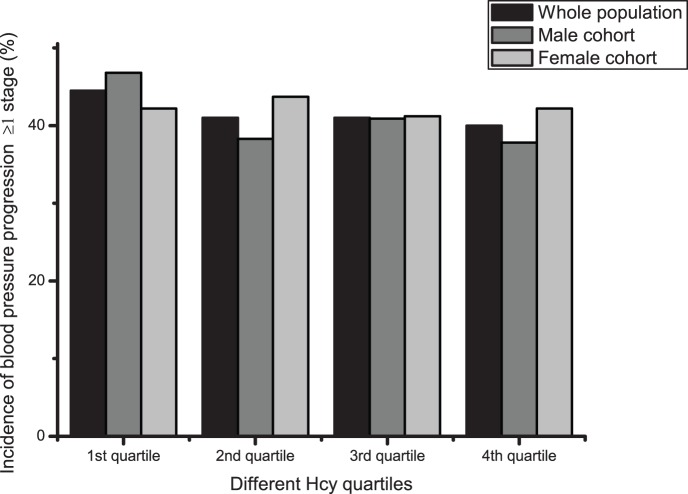
Blood pressure progression incidences within different Hcy quartiles. Black bar: Whole population; Dark gray bar: Male cohort; Light grey bar: Female cohort.

**Figure 3:**
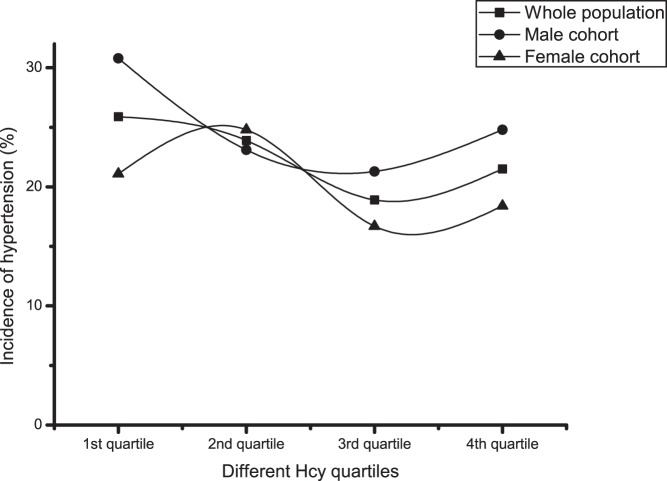
Incidence of hypertension within different Hcy quartiles. Square symbol: Whole population; Circle symbol: Male cohort; Up-triangle symbol: Female cohort.

**Figure 4:**
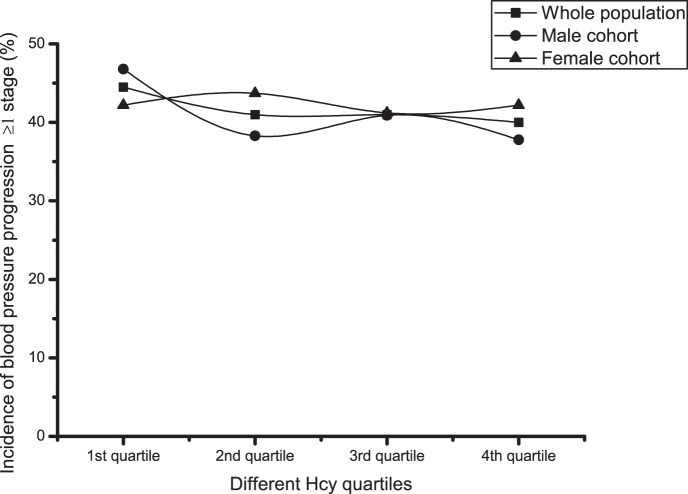
Blood pressure progression incidences within different Hcy quartiles. Square symbol: Whole population; Circle symbol: Male cohort; Up-triangle symbol: Female cohort.

The study population included cases who had not experienced cerebral infarction or myocardial infarction and were aged 40 years or older. The distribution of Hcy was skewed, probably due to age. For the skewedness distribution of Hcy, the data were logarithmically transformed before being grouped into quartiles (Table 2). Of 2427 enrolled subjects, 557 subjects (22.5%) had hypertension after 2 years of follow-up, with an incidence of 24.9% and 20.2% in the male and female cohorts, respectively. For both the whole group and the male cohort, the hypertension incidence distributions were approximately U-shaped (*P*<0.05), but were not statistically significant (*P* = 0.065) for the female cohort. Of the overall cohort, 1029 subjects (41.6%) had a blood pressure progression of more than one stage, with an incidence of 40.9% and 42.3% in the male and female cohorts, respectively. There were no statistically significant differences between the quartiles for blood pressure progression (Table 2 and Figure 1 to 4).

**Table 2:** Hypertension and blood pressure progression incidences within different Hcy quartiles.

Characteristics		1st quartile	2nd quartile	3rd quartile	4th quartile	*P*-value
**Cases**		607	619	624	622	
**Hcy levels**	**Whole population**	6.4 (4.9, 9.8)	9.8 (8.2, 13.4)	13 (11.2, 18)	23.4 (16.5, 30.7)	
	**Male cohort**	8.9 (7.5, 10.2)		13.35 (12.5, 14.5)	18 (16.7, 19.6)	
	**Female cohort**	5.1 (3.9,6)	8.2 (7.5, 9.1)	11.2 (10.5, 12)	16.6 (14.7, 21.1)	
**Incidence of hypertension**	**Whole population**	157 (25.9)	148 (23.9)	118 (18.9)	134 (21.5)	**0.023**
	**Male cohort**	92 (30.8)	71 (23.1)	64 (21.3)	76 (24.8)	**0.042**
	**Female cohort**	65 (21.1)	77 (24.8)	54 (16.7)	58 (18.4)	0.065
**Incidence of blood pressure progression ≥1 stage**	**Whole population**	270 (44.5)	254 (41)	256 (41)	249 (40)	0.412
	**Male cohort**	140 (46.8)	118 (38.3)	123 (40.9)	116 (37.8)	0.093
	**Female cohort**	130 (42.2)	136 (43.7)	133 (41.2)	133 (42.2)	0.934

### Risk of Hypertension and Blood Pressure Progression ≥1 Stage by Hcy Level

Taking incident hypertension or blood pressure progression ≥1 stage as the dependent variable and Hcy quartiles, lg(−Hcy), or Hcy ≥16 µmol/L as independent variables, unconditional logistical regression was applied to assess the risk of blood pressure progression by Hcy level. Model 1 was univariable analyses, model 2 was adjusted for age and gender, and model 3 was a multivariable model further adjusted for other confounding characteristics, including baseline BMI, waistline, SBP, DBP, FBG, TG, TC, HDL, LDL, UA, CR, smoking, drinking, exercise, and salt status. Compared with the third quartile, and after adjusting for potential confounding factors, the risk of incident hypertension in the first and second quartile was increased (*P*<0.05), and the odds ratio (OR) was 1.55 (95% CI 1.154–2.081) and 1.501 (95% CI 1.119–2.013), respectively. No statistically significant relationship was found for Hcy quartiles with blood pressure progression ≥1 stage for either lg(−Hcy) or Hcy ≥16 µmol/L with incident hypertension or blood pressure progression ≥1 stage (Table 3). We then added TC, TG, LDL-C, blood glucose, diabetes, BMI, obesity, and exercise, respectively, to the model as interaction variables with Hcy, No interaction was observed between these risk factors and Hcy (Table 4).

**Table 3:** Risk of Hypertension and Blood Pressure Progression ≥1 Stage by Hcy Level.

			Hypertension		Blood pressure progressed ≥1 stage	
			OR (95% CI)	*P*-Value	OR (95% CI)	*P*-Value
**Quartiled Hcy levels (3rd quartile as control)**	**Model 1**	**3rd quartile**	1	0.023	1	0.413
		**1st quartile**	1.496 (1.142–1.961)	0.004	1.152 (0.919–1.444)	0.221
		**2nd quartile**	1.347 (1.026–1.77)	0.032	1 (0.798–1.254)	0.998
		**4th quartile**	1.177 (0.893–1.553)	0.248	0.96 (0.765–1.203)	0.721
	**Model 2**	**3rd quartile**	1	0.004	1	0.23
		**1st quartile**	1.609 (1.222–2.118)	0.001	1.198 (0.953–1.505)	0.122
		**2nd quartile**	1.398 (1.062–1.84)	0.017	1.022 (0.814–1.282)	0.854
		**4th quartile**	1.154 (0.874–1.525)	0.313	0.951 (0.758–1.193)	0.663
	**Model 3**	**3rd quartile**	1	0.004	1	0.447
		**1st quartile**	1.598(1.186−2.155)	0.002	1.146(0.891−1.475)	0.288
		**2nd quartile**	1.540(1.144−2.073)	0.004	1.132(0.883−1.452)	0.329
		**4th quartile**	1.142(0.844−1.545)	0.391	0.966(0.752−1.241)	0.787
**Lg(−** **Hcy)**	**Model 1**		1.08 (0.753–1.55)	0.675	0.998 (0.735–1.354)	0.988
	**Model 2**		0.737 (0.489–1.111)	0.145	1 (0.708–1.413)	1
	**Model 3**		0.69(0.445−1.068)	0.096	1.045(0.709−1.54)	0.825
**Hcy ≥16 µmol/L**	**Model 1**		1.114 (0.909–1.365)	0.299	1.012 (0.85–1.204)	0.896
	**Model 2**		0.95 (0.762–1.186)	0.652	1.01 (0.836–1.22)	0.916
	**Model 3**		0.866(0.68−1.103)	0.245	0.945(0.762−1.172)	0.605

Model 1: Univariable Logistic Regression; Model 2: Adjusted for age and gender; Model 3: Adjusted for age, gender, BMI, waistline, SBP, DBP, FBG, TG, TC, HDL, LDL, UA, CR, smoking, drinking, exercise and salt status**.**

FBG: Fast blood glucose; TG: Triglyceride; TC: Total cholesterol; HDL-C: High-density lipoprotein cholesterol; LDL-C: Low-density lipoprotein cholesterol; BMI: Body mass index; UA: uric acid; CR: creatinine; Hcy: homocysteine.

**Table 4:** Interactions Between Related Risk Factors and Hcy.

	TC	TG	LDL-C	exercise	blood glucose	diabetes	BMI	obesity
**P value**	**0.128**	**0.335**	**0.409**	**0.871**	**0.15**	**0.103**	**0.594**	**0.587**

### Risk of Hypertension Based on Hcy Between Genders

Because the distribution of hypertension incidence was different between males and females, we analyzed the risk of hypertension by Hcy quartiles in the two sex cohorts. The results showed that compared with the third quartile, the risk of the first and second quartile was increased (*P*<0.05) in the male cohort (OR 1.80 [95% CI 1.190–2.723] and 1.533 [95% CI 1.002–2.347], respectively), whereas no statistically significant relationship was found in the female cohort (Table 5).

**Table 5:** Risk of Hypertension by Hcy Level Between Genders.

		Male		Female	
		OR (95% CI)	*P*-Value	OR (95% CI)	*P*-Value
**Model 1**	**3rd quartile**	1	0.043	1	0.067
	**1st quartile**	1.646(1.137−2.382)	0.008	1.332(0.893−1.989)	0.16
	**2nd quartile**	1.109(0.757−1.627)	0.595	1.639(1.11−2.42)	0.013
	**4th quartile**	1.218(0.834−1.779)	0.307	1.124(0.747−1.691)	0.574
**Model 2**	**3rd quartile**	1	0.008	1	0.061
	**1st quartile**	1.862(1.276−2.717)	0.001	1.35(0.901−2.023)	0.145
	**2nd quartile**	1.196(0.812−1.761)	0.366	1.648(1.115−2.434)	0.012
	**4th quartile**	1.223(0.835−1.791)	0.302	1.114(0.739−1.678)	0.606
**Model 3**	**3rd quartile**	1	0.041	1	0.05
	**1st quartile**	1.8(1.19−2.723)	0.005	1.56(1.001−2.43)	0.049
	**2nd quartile**	1.533(1.002−2.347)	0.049	1.607(1.049−2.461)	0.029
	**4th quartile**	1.312(0.865−1.99)	0.202	1.031(0.656−1.62)	0.895

## Discussion

In the present study, we investigated the association between serum Hcy level and incidence of hypertension in a community-based cohort during 2 years of follow-up. Interestingly, we found an approximately U-shaped distribution of the incidence of hypertension with increasing Hcy level quartiles. Moreover, in the male cohort, low Hcy level might serve as a risk factor of the disease.

The relationship between HHcy and hypertension has been proposed by multiple researchers, but the causal effect of HHcy on hypertension did not reach a consensus as many of these results were obtained in cross-sectional studies [16], [17]. In the past decade, several prospective studies have focused on this topic; however, the results were ambiguous. In a 4-year follow-up study in 2104 Framingham Heart Study participants [18], no major relationship between baseline plasma Hcy levels and incidence of hypertension or longitudinal blood pressure progression was found. In 2006, another nested case-control study with 17.5 years of follow up also demonstrated that men with higher Hcy levels had an increased but not statistically significant risk of incident hypertension [19]. A similar result was seen in other prospective studies of multiple biomarkers for the risk of incident hypertension [20], [21]. These results indicated that there was possibly no significant causal relationship between HHcy and hypertension [22]. Collateral controversial evidence [23] came from research showing that higher folate intake during young adulthood was longitudinally associated with a lower incidence of hypertension later in life, but the Hcy levels of subjects in this study were not discussed. Differing from the above results, in this longitudinally-designed study we observed that the incidence of hypertension in the third quartile was the lowest, which was different from the increasing incidence found in the Framingham Study [18]. Here, we found an approximately U-shaped distribution of incident hypertension risk by Hcy, and this distribution was more evident in the male population. Additionally, we also noted that the mean Hcy levels in the Framingham study were much lower (nearly equal to the first three quartiles in the present study), especially in male subjects, further strengthening the difference between the two studies.

After adjusting for the possible confounding factors in logistical regression, the approximate U-shaped distribution of risk was still statistically significant. Compared with the third quartile, the risk of incident hypertension in the first and second quartiles was increased by 1.55 and 1.50 fold, respectively, whereas the forth quartile was not significant. These results led to the conclusion that rather than HHcy, lower Hcy levels might be a risk factor for incident hypertension. Similar results were observed in Bowman’s study among male physicians [19]. Although the risk of incident hypertension seemed to increase with higher Hcy levels, we saw an obvious decrease in the fourth quartile, which was also distributed in an approximately U-shaped curve. These results indicate that the causal relationship between HHcy and hypertension might not be simple or absolute, and that there might be an optimal Hcy level existing at relatively higher levels for the prevention of hypertension. Recently, several studies on Hcy responses to anti-hypertensive therapy were completed, but these results were also conflicting[24]–[26]. In a study on the effect of enalapril, a significant increase in plasma Hcy levels (*P* = 0.02) among hypertensive patients with Hcy <10 mmol/L was observed, whereas there was no change in patients with Hcy >10 mmol/L [25]. The mechanism and possible underlying relationship with our results still needs further study.

Hcy was regarded as an indicator of oxidative stress status. During HHcy, increased reactive oxygen species, matrix metalloproteinase, and decreased endothelial nitric oxide cause vascular constriction and stiffness, which could lead to essential hypertension [27]. A clinical study came to the same conclusion, that Hcy was a determinant of vascular thickness and compliance in hypertensive patients [28], [29] and that HHcy increased the risk of cerebrovascular complications and glomerular sclerosis [30], [31]. There is a pathogenic effect of Hcy on the vasculature, but the data on the relationship between Hcy levels and vascular biology at the initial stages of hypertension was limited. The results of our study suggest that at an initial stage of hypertension, lower Hcy levels might indicate higher risk of the disease. The Hcy-related vascular lesion should be further investigated.

A gender difference was also noted in the present study. Females had lower Hcy levels compared with males, and Hcy was not a significant risk factor for females. This difference might be due to female hormones, which were demonstrated to have antioxidant effects that may antagonize the risk based on Hcy [32]. Further studies are needed to investigate the mechanism, but gender is an important factor that should be considered in future studies on the relationship between Hcy and hypertension.

This study has some limitations. Firstly, the 2-year follow-up period might be insufficient, and could lead to bias in underestimating the influence of Hcy on the disease. However, as mentioned above, a similar approximately U-shaped curve for risk was found in a 17.5-year follow-up study [33], and more research or meta-analyses are needed to assess any potential bias. From 2010 to the present, we measured only two blood biochemical parameters. Although the follow-up time was only two years, we also came to the conclusion that the effect of HCY on the incidence of hypertension. The fifth physical examination will occur in 2014, and we will continue to focus on the impact of Hcy on the incidence of hypertension. Secondly, serum folate and vitamin B, which are known to influence Hcy levels, were not evaluated in this study. Thirdly, serum Hcy levels in some of the 2012–2013 physical examinations were missing. Fourthly, we have not completed an analysis of genetic susceptibility for hypertension in our study population.

## Conclusion and Perspectives

In this longitudinal study, we found an approximately U-shaped risk distribution of Hcy levels for incident hypertension, especially in males. Other than the increasing risk derived from cross-sectional studies and the non significant association found later in several prospective studies, we hypothesize that HHcy might not be simply related to the increasing risk of incident hypertension; moreover, mildly elevated Hcy levels (about 15 µmol/L) might be a protective factor in males.

From the point of view of this study, caution should be used when considering Hcy levels to guide dietary supplementation or therapy aimed at decreasing the risk of hypertension. Simply lowering Hcy levels might not decrease the risk, or even lead to a reverse effect in some specific populations. However, we should also be aware that besides lowering Hcy levels, dietary supplementation with folate or vitamin B, both of which are in the Hcy metabolism pathway, provide additional benefits beyond the risk of incident hypertension. Rather than deny these benefits, the results of this study remind us of the possibility that there are other mechanisms that may explain how Hcy may be involved in vascular pathology.
